# Supplementary material for: End Sequence Analysis Toolkit (ESAT) expands the extractable information from single-cell RNA-seq data
Source: Genome Res. 2016 Oct;26(10):1397–410. doi: 10.1101/gr.207902.116 (PMC5052061; doi:10.1101/gr.207902.116)
Supplement: Supplemental Material [file supp_gr.207902.116_Supp_Materials.tar.gz › ESAT-esat_v0.1/doc/html/interfacebroad_1_1core_1_1hmm_1_1_markov_state_3_01_t_01_4.html]

ESAT: broad.core.hmm.MarkovState< T > Interface Reference


ESAT


- Main Page
- Related Pages
- Packages
- Classes
- Files

- Class List
- Class Index
- Class Hierarchy
- Class Members

All Classes Namespaces Files Functions Variables Enumerator Pages

- broad
- core
- hmm
- MarkovState< T >

Public Member Functions |
List of all members

broad.core.hmm.MarkovState< T > Interface Reference

|  |  |
| --- | --- |
| Public Member Functions | |
| double | getEmissionProbability (T observation) |
|  | |
| String | getName () |
|  | |
| double | getEmissionLogProbability (T observation) |
|  | |
| T | emitObservation () |
|  | |

## Member Function Documentation

|  |  |  |  |  |
| --- | --- | --- | --- | --- |
| T broad.core.hmm.MarkovState< T >.emitObservation | ( |  | ) |  |

Uses its background distribution to emit a sampled observation.

Returns

|  |  |  |  |  |  |
| --- | --- | --- | --- | --- | --- |
| double broad.core.hmm.MarkovState< T >.getEmissionLogProbability | ( | T | *observation* | ) |  |

In many cases the implementing class may be able to implement this method so that the logarithm is not on every call.

Parameters
:   |  |  |
    | --- | --- |
    | observation | The roll of a dice, an alignment column, a nucleotide, etc. |

Returns
:   natural log of P(observation | this state)

|  |  |  |  |  |  |
| --- | --- | --- | --- | --- | --- |
| double broad.core.hmm.MarkovState< T >.getEmissionProbability | ( | T | *observation* | ) |  |

Parameters
:   |  |  |
    | --- | --- |
    | observation | The roll of a dice, an alignment column, a nucleotide, etc. |

Returns
:   P(observation | this state)

|  |  |  |  |  |
| --- | --- | --- | --- | --- |
| String broad.core.hmm.MarkovState< T >.getName | ( |  | ) |  |

Returns
:   The name of this Markov State

Here is the caller graph for this function:

---

The documentation for this interface was generated from the following file:

- java/broad/core/hmm/MarkovState.java


---

Generated on Fri Aug 15 2014 15:10:52 for ESAT by  

 1.8.7
